# Abdominal Compartment Syndrome Caused by Massive Pyonephrosis in an Infant with Primary Obstructive Megaureter

**DOI:** 10.1155/2011/174167

**Published:** 2011-07-31

**Authors:** Silvia K. Kavaguti, Barbara R. Mackevicius, Murilo F. de Andrade, Silvio Tucci Jr, Ana P. C. P. Carlotti

**Affiliations:** ^1^Division of Pediatric Critical Care Medicine, Department of Pediatrics, Hospital das Clínicas, Faculty of Medicine of Ribeirão Preto, University of São Paulo, Avenida dos Bandeirantes 3900, 14049-900 Ribeirão Preto, SP, Brazil; ^2^Division of Urology, Department of Surgery and Anatomy, Hospital das Clínicas, Faculty of Medicine of Ribeirão Preto, University of São Paulo, Avenida dos Bandeirantes 3900, 14049-900 Ribeirão Preto, SP, Brazil

## Abstract

The authors report a case of abdominal compartment syndrome caused by massive pyonephrosis in an infant with primary obstructive megaureter successfully treated with emergency surgical decompression.

## 1. Introduction

Abdominal compartment syndrome (ACS) is associated with high mortality if unrecognized and untreated [1–3]. It may be caused by any condition that increases the volume of the abdominal or retroperitoneal contents and thereafter, the intra-abdominal pressure (IAP) [4]. Increased IAP may affect multiple organ systems, including respiratory, cardiovascular, renal, gastrointestinal, hepatic, and central nervous system [4, 5]. The most common clinical manifestations are respiratory failure with progressive hypoxia and hypercapnia, elevated airway pressures in mechanically ventilated patients, oliguria, reduced cardiac output, and venous stasis. Signs of impaired liver and gut perfusion and raised intracranial pressure may also be observed [5]. Early recognition of patients at risk of developing intra-abdominal hypertension and timely intervention to avoid the progression to multisystem organ failure are of paramount importance to minimize the morbidity and mortality of ACS. Pyonephrosis is a rare condition in children that may complicate urinary tract obstruction. We report a case of massive pyonephrosis leading to ACS with marked improvement after surgical decompression. To our knowledge, this is the first case of ACS caused by massive pyonephrosis reported in the literature.

## 2. Case Presentation

**Figure 1:**
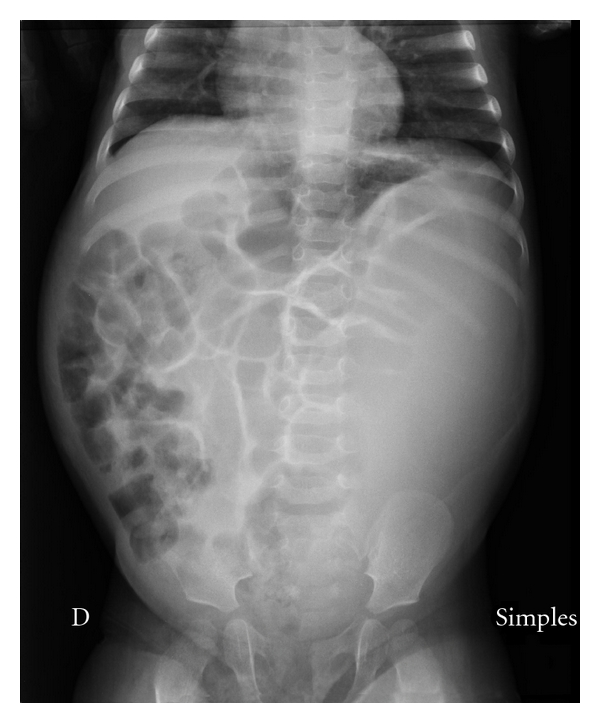
Abdominal plain radiography showing elevation of both hemidiaphragms, prominent gaseous distension of bowel loops, which are completely deviated to the right side of the abdomen, and homogeneous opacity of the left hemiabdomen.

**Figure 2:**
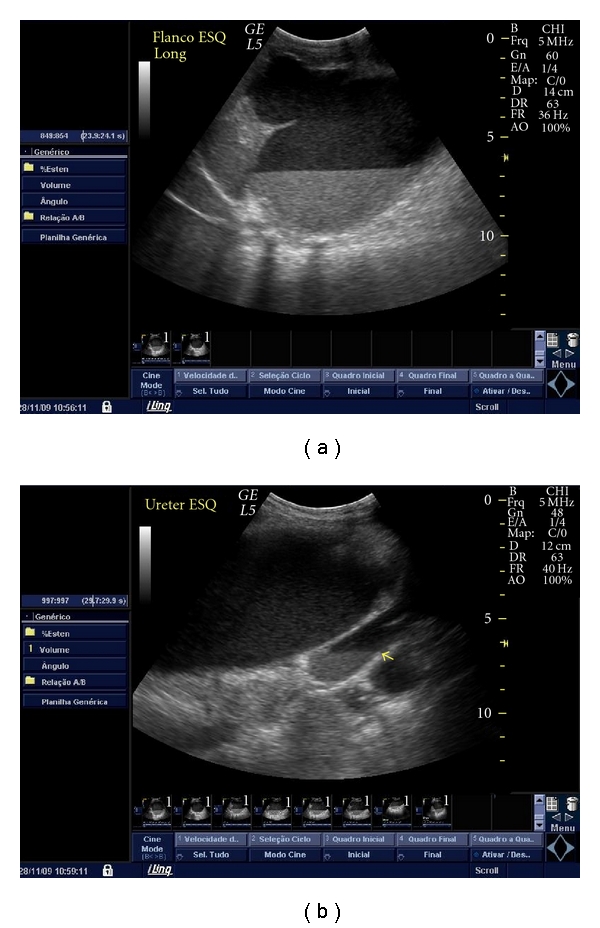
Abdomen ultrasound showing marked dilatation of the left pelvicalyceal system (a) and left ureter ((b) arrow), both containing hypoechoic fluid with posterior acoustic enhancement, and echogenic material deposited on the gravity-dependent portion with formation of fluid-fluid level.

**Figure 3:**
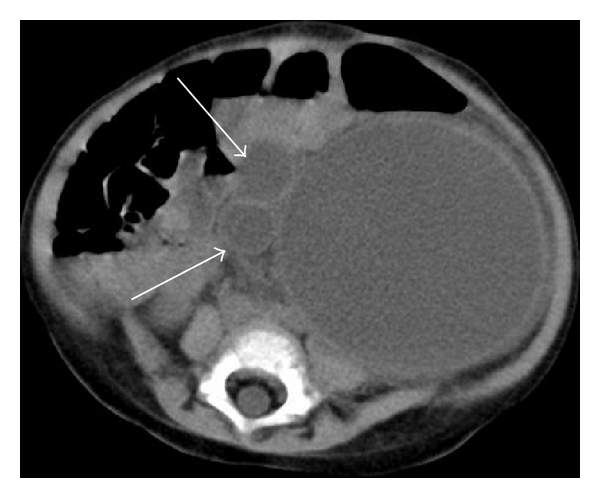
Computed tomographic scan of the abdomen showing a rounded appearance of the abdomen, markedly dilated and tortuous left ureter (arrows), and severe pelvicalyceal dilatation.

An 11-month-old female was referred to our hospital because of severe abdominal distension, lethargy and poor feeding. She had developed a progressively distended abdomen in the course of 5 days, with fever, constipation, decreased urine output, and increasing respiratory distress. Her past medical history was remarkable for a prenatal ultrasonographic diagnosis of primary obstructive megaureter on the left side and poor parental compliance in administering prophylactic antibiotics at home. On physical exam at pediatric intensive care unit (PICU) admission, the patient appeared obtunded and febrile, with moderate respiratory distress. Her vital signs were as follows: temperature 38°C, respiratory rate 72 breaths/min, heart rate 180 beats/min, and blood pressure 119/72 mm Hg. Respirations were rapid and shallow. Nasal flaring and moderate subcostal retractions were noted. Her central and peripheral pulses were strong, with capillary refill time < 2 sec in upper extremities and 4 sec in lower extremities. Both lower limbs had a mottled cyanotic appearance. Abdominal examination revealed marked distension with diminished bowel sounds and moderate discomfort with deep palpation of the left upper quadrant and left flank. Signs of peritoneal irritation were absent. A 10 × 5 cm firm abdominal mass was palpable from the left costal margin to the left iliac fossa. Intra-abdominal pressure measured by the intravesical route was 14 mm Hg. Laboratory investigations revealed a hemoglobin of 7.4 g/dL, platelets of 695000/mm^3^, and white blood cell (WBC) count of 10600/mm^3^ (13% bands, 28% neutrophils, 54% lymphocytes, 4% eosinophils, 3% monocytes); the neutrophils showed toxic granulation and vacuolation. C-reactive protein was 28.3 mg/dL, and arterial blood gas analysis (while the patient was breathing room air) revealed a pH of 7.38, PCO_2_ of 28 mm Hg, PO_2_ of 67 mm Hg, and bicarbonate of 16 mEq/L. Arterial lactate was 1.4 mmol/L, serum urea concentration was 25 mg/dL, and serum creatinine concentration was 0.3 mg/dL. Urine was cloudy, and her urine output was 0.3 mL/kg/h. Urinalysis showed 15–20 WBC per high-power field and 6–8 red blood cells per high-power field, with 1+ protein, a pH of 5, and a negative nitrite test. Chest radiography showed elevated hemidiaphragms, with reduced lung expansion. Abdominal plain radiography showed prominent gaseous distension of bowel loops which were completely deviated to the right side of the abdomen and homogeneous opacity of the left hemiabdomen (Figure 1). Abdomen ultrasound revealed marked dilatation of calyces, pelvis and ureter on the left side, and ureter and collecting system containing hypoechoic fluid with posterior acoustic enhancement, and echogenic material deposited on the gravity-dependent portion with formation of fluid-fluid level (Figure 2). The right kidney and the right collecting system were normal. Computed tomography showed a rounded appearance of the abdomen, a markedly dilated and tortuous left ureter, and severe pelvicalyceal dilatation with increased renal size and parenchymal thinning (Figure 3). Antibiotic therapy with cefotaxime was started, and emergency surgical decompression was performed. The patient underwent a left pyelostomy through a dorsal lumbotomy incision, which revealed a large amount of pus, and after drainage of this, her condition greatly improved. In the immediate postoperative period, she had a respiratory rate of 31 breaths/min, heart rate of 134 beats/min, and blood pressure of 116/60 mm Hg, with symmetric pulses and capillary refill time < 2 sec in upper and lower extremities. No abdominal mass was palpable, and IAP was 3 mm Hg. Her urine output increased to 3.5 mL/kg/h promptly. Because urine culture obtained by urinary catheter yielded more than 10^5^ colony-forming units per mL of *Klebsiella pneumoniae*, amikacin was added to the antibiotic regimen. She made a complete recovery and was discharged from the PICU on the 5th day postadmission and from the hospital 8 days later, with follow-up care in the pediatric nephrology outpatient clinic.

## 3. Discussion

Because signs and symptoms of ACS are often subtle, its diagnosis depends on a high index of suspicion in patients who acutely develop tense abdominal distension. In our patient, ACS manifested as oliguria, respiratory insufficiency with hypoxia, and venous congestion of the lower limbs and ileus. Nevertheless, given the low sensitivity of the physical examination alone (40–60%) for the detection of increased IAP [6, 7], it is also important to perform serial IAP measurements in patients at risk of developing ACS [8, 9]. The International Conference of Experts on Intra-abdominal Hypertension and Abdominal Compartment Syndrome proposed that ACS be defined as a sustained increase of IAP greater than 20 mm Hg (with or without abdominal perfusion pressure—calculated as mean arterial pressure minus IAP—lower than 60 mm Hg) associated with new organ dysfunction or failure [1]. Due to its simplicity and low cost, measurement of bladder pressure has been considered the gold standard method for determining IAP [8]. However, the level of IAP at which ACS occurs in children has not been definitely established because children may exhibit organ dysfunction with lower IAP values [10, 11]. In our patient, ACS developed at an IAP of 14 mm Hg. Therefore, a more appropriate definition of ACS in children is needed. It has recently been suggested that it may be more convenient to define ACS as the development of new organ dysfunction associated with a rising or sustained elevation in IAP, regardless of the actual IAP value [12]. 

Obstructed renal collecting systems are at risk for superimposed infections. Our patient had an obstructive urinary tract malformation which complicated with pyonephrosis. Of note, she was not receiving prophylactic antibiotics because of poor parental compliance with treatment. In a cohort of 49 children with primary obstructive megaureter, urinary tract infection was the most common complication, and its incidence in children less than 1 year of age was 55% lower during antibiotic prophylaxis [13]. 

Delayed diagnosis and treatment of pyonephrosis have been associated with irreversible damage to the kidneys and the development of septic shock [14]. However, the mass effect of massive pyonephrosis resulting in increased IAP and ACS has not been previously reported. In patients with ACS, early surgical decompression of the abdomen has been associated with a marked improvement in organ function [10, 15–18]. In fact, the indications for emergency surgery in our patient were twofold: first, abdominal decompression to prevent the progression to multiple organ failure and second, drainage of the collection of pus to minimize the risk of septic shock. In critically ill patients, many factors other than increased IAP can produce several of the clinical signs and symptoms described in patients with intra-abdominal hypertension and ACS and may contribute to the development of multiple organ failure. Our patient had signs and symptoms that could suggest sepsis/septic shock (tachycardia, tachypnea, fever, decreased urine output and obtundation). However, following emergency surgical decompression, the patient had a prompt normalization of her vital signs and urine output, coincident with a fall in IAP, which argues against the only diagnosis of sepsis/septic shock. As a matter of fact, it is well known that timely surgical decompression of the abdomen produces an immediate improvement of cardiopulmonary and renal function, differently from what occurs in patients with septic shock whose organ dysfunction takes several hours to days to improve after initiation of treatment.

One alternative treatment to open pyelostomy is percutaneous nephrostomy. Nevertheless, because the patient parents had been poorly compliant with treatment before, the surgical team opted for an open pyelostomy as it does not require the maintenance of an indwelling catheter. 

In conclusion, massive pyonephrosis complicating urinary tract obstruction can cause ACS which may be successfully treated by timely surgical drainage and abdominal decompression. This case illustrates the importance of awareness, early diagnosis, and management of ACS to avoid the progression to multiple organ failure, thus resulting in improved outcome.
